# Graphene-Based Composites for Biomedical Applications: Surface Modification for Enhanced Antimicrobial Activity and Biocompatibility

**DOI:** 10.3390/biom13111571

**Published:** 2023-10-24

**Authors:** Rita Teixeira-Santos, Samuel Belo, Rita Vieira, Filipe J. M. Mergulhão, Luciana C. Gomes

**Affiliations:** 1 LEPABE—Laboratory for Process Engineering, Environment, Biotechnology and Energy, Faculty of Engineering, University of Porto, Rua Dr. Roberto Frias, 4200-465 Porto, Portugal; up201608956@edu.fe.up.pt (S.B.); up201603193@edu.fe.up.pt (R.V.); filipem@fe.up.pt (F.J.M.M.); luciana.gomes@fe.up.pt (L.C.G.); 2 ALiCE—Associate Laboratory in Chemical Engineering, Faculty of Engineering, University of Porto, Rua Dr. Roberto Frias, 4200-465 Porto, Portugal

**Keywords:** graphene-based materials, surface modification, antimicrobial activity, biocompatibility, biomedical applications

## Abstract

The application of graphene-based materials in medicine has led to significant technological breakthroughs. The remarkable properties of these carbon materials and their potential for functionalization with various molecules and compounds make them highly attractive for numerous medical applications. To enhance their functionality and applicability, extensive research has been conducted on surface modification of graphene (GN) and its derivatives, including modifications with antimicrobials, metals, polymers, and natural compounds. This review aims to discuss recent and relevant studies related to advancements in the formulation of graphene composites, addressing their antimicrobial and/or antibiofilm properties and evaluating their biocompatibility, with a primary focus on their biomedical applications. It was concluded that GN surface modification, particularly with compounds intrinsically active against bacteria (e.g., antimicrobial peptides, silver and copper nanomaterials, and chitosan), has resulted in biomaterials with improved antimicrobial performance. Furthermore, the association of GN materials with non-natural polymers provides composites with increased biocompatibility when interfaced with human tissues, although with slightly lower antimicrobial efficacy. However, it is crucial to highlight that while modified GN materials hold huge potential, their widespread use in the medical field is still undergoing research and development. Comprehensive studies on safety, long-term effects, and stability are essential before their adoption in real-world medical scenarios.

## 1. Introduction

In recent years, graphene materials have attracted significant interest due to their remarkable properties and various applications. Graphene (GN) is a two-dimensional carbon allotrope composed of a single layer of carbon atoms arranged in a hexagonal lattice [1]. This structure, which has a high surface area and large aspect ratio, confers high electronic and thermal conductivities to GN, as well as superior mechanical strength [2,3,4]. In addition, GN exhibits a high ability to interact with other molecules through various processes, including physical and chemical interactions [5].

Graphene derivatives can be generated by introducing oxygen-containing functional groups to the GN structure or reducing its oxide form, obtaining graphene oxide (GO) or reduced graphene oxide (rGO) [6], respectively. Additionally, GN monolayers can be modified with metals, antimicrobial drugs, polymers, and natural compounds [7,8,9,10]. While these derivatives preserve their original properties, they also offer enhanced advantages, such as improving the GN dispersion factor in solvents or polymeric matrices and reducing GN toxicity [11,12,13]. As a result, GN has been applied in numerous industries, including construction [14], energy [15], food [16], environmental [17], and biomedical [18]. 

Within the medical field, there have been notable technological advances in the application of GN and its derivatives in drug/gene delivery, biosensing, bioimaging [19], wound healing [20], and tissue engineering [18]. Furthermore, due to the antimicrobial activity and biocompatibility of GN-based materials, they are deemed suitable for manufacturing implantable medical devices such as cardiovascular stents, orthopaedic scaffolds, and urinary implants [21,22].

**Figure 1:**
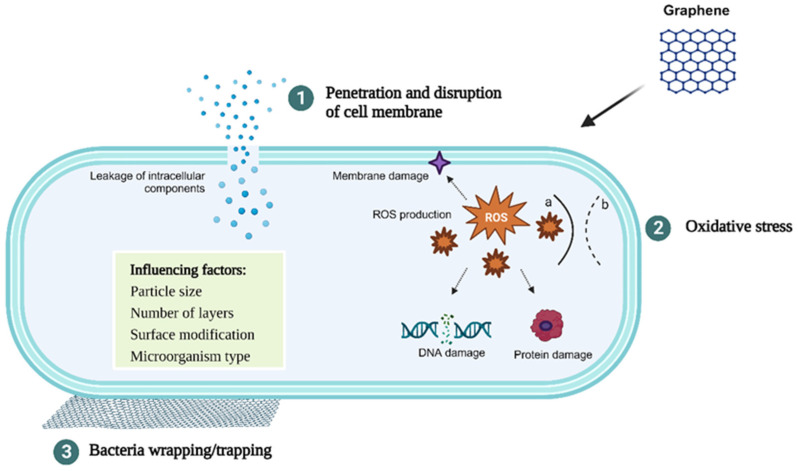
Schematic representation of the antibacterial mechanisms of GN and factors influencing its antimicrobial activity. (1) Penetration and disruption of the bacterial cell membrane with consequent leakage of the intracellular content; (2) oxidative stress with (a) and without (b) generation of reactive oxygen species (ROS); (3) bacteria wrapping/trapping.

To date, different mechanisms have been proposed to explain the antimicrobial activity of GN and its derivatives (Figure 1). GN’s sharp edges can physically damage bacterial cell membranes, leading to loss of their integrity, leakage of intracellular content, and, ultimately, cell death [23,24,25]. GN can also generate oxidative stress, which may come from different paths—reactive oxygen species (ROS)-dependent or ROS-independent pathways—which, in either case, disrupt cellular functions, resulting in cell inactivation [25,26]. Lastly, GN-based materials can also wrap and trap bacterial cells [26,27,28]. GN can act as a barrier that traps and isolates bacteria from the environment, further inhibiting their proliferation. Furthermore, this wrapping/trapping effect induces cell membrane damage in combination with the other mechanisms mentioned previously [28]. The interaction between GN and bacterial cell membranes is believed to be the driving factor behind the toxicity exerted by these carbon materials.

The antimicrobial potential of GN is related not only to its structural properties (e.g., particle size and number of layers) but also to the surface modification, the nature of the targeted microorganism (Gram-positive or Gram-negative bacteria), and the environment where GN and microbial cells interact (Figure 1).

Smaller GN particles with a higher surface-to-volume ratio can interact more effectively with microbial cells and affect their membrane integrity [29,30]. In addition, smaller particles may diffuse more easily within microbial biofilms, allowing better disruption of their structure and function [31]. Likewise, few-layer GN sheets have shown a strong antimicrobial effect, leading to significant damage to microbial cell membranes [32]. Contrarily, more layers decrease the GN dispersibility and its contact with microorganisms [26]. Previous studies have also reported that GN modified with cationic functional groups is more toxic to microbial cells than GN containing neutral or negatively charged groups [33,34]. This may be attributed to favorable electrostatic interactions between the positively charged GN surfaces and negatively charged microbial cell membranes [34]. Functionalization can also change the GN surface hydrophobicity [33], thereby enhancing its dispersibility [35]. Furthermore, GN surface modification allows the attachment of bioactive molecules, such as antimicrobial agents or peptides, which specifically target and disrupt microbial cells [8,36].

Studies have revealed that GN and its derivatives, such as GO and rGO, can inactivate both Gram-positive and Gram-negative bacteria in either planktonic or sessile states [37]. However, it has been evidenced that these graphene-based materials are more effective towards Gram-positive than Gram-negative bacteria [38,39]. While Gram-positive bacteria contain a plasmatic membrane and a thick peptidoglycan layer, Gram-negative bacteria have an outer membrane mainly composed of lipopolysaccharides, which may offer additional protection against chemical and physical stress [40]. Additionally, GN materials have shown promising antimicrobial activity against drug-resistant bacteria; therefore, they can be considered for therapeutic purposes [41]. 

When using GN materials for medical applications, it is crucial to comprehensively evaluate their biocompatibility to ensure that they are safe for use in real scenarios, such as medical devices or wound dressings. Numerous studies have been conducted and the results are mostly promising [27,42,43,44]. In vitro studies have shown that GN-based materials can interact with human cells, and their effects on cell viability and proliferation can vary depending on factors like GN size, concentration, and surface functionalization [45,46]. In general, lower concentrations and well-dispersed GN tend to be more biocompatible. Likewise, surface functionalization plays a significant role in increasing GN biocompatibility [45].

**Figure 2:**
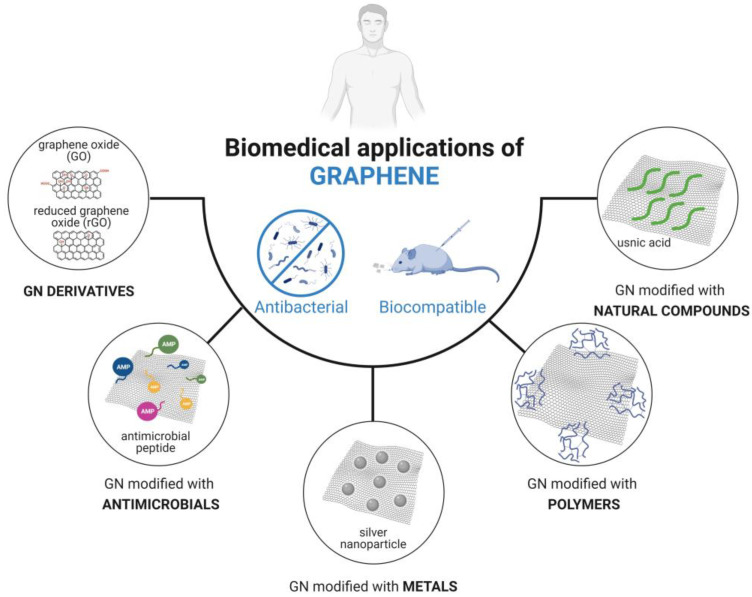
Types of graphene (GN) modifications discussed in this review for potential use in the biomedical field.

Despite the plentiful range of GN-based materials developed in recent years, most are still far from practical biomedical applications. As a result, further investigation is necessary to understand the functionality, applicability, and safety of these carbon materials. Hence, this review aims to critically discuss recent progress in the formulation of graphene composites, assessing their antimicrobial and/or antibiofilm activities while also evaluating their biocompatibility, with a focus on biomedical applications. Research involving GN and its derivatives modified with antimicrobials, metals, polymers, or natural compounds is addressed (Figure 2). By summarizing and categorizing recently developed GN-based composites based on their surface modification type, this review provides a comprehensive perspective on the applicability and effectiveness of GN materials in the medical field, including insights into GN–bacteria interactions. Additionally, it serves as a valuable resource to aid researchers in the development and optimization of GN materials tailored for specific medical applications.

## 2. Graphene Modified with Antimicrobials

One desirable feature of graphene is its capacity to bind to a variety of molecules, including antimicrobial agents, peptides, and biocides. This capability not only broadens the range of potential applications of GN materials but also has the potential to enhance their antimicrobial activity and biocompatibility [47]. Table 1 summarizes recent studies that assessed the biocompatibility and antimicrobial performance of GN materials modified with antimicrobial compounds, including antibiotics [48], antimicrobial peptides [8,49,50], and disinfectants [36]. 

Tran et al. [48] modified the GO surface with doxycycline (Dox), a bacteriostatic antibiotic, and coated titanium (TiO_2_) surfaces for potential application in medical devices. The results demonstrated that Dox-modified GO/TiO_2_ surfaces reduced the viability of adhered bacteria by 90% compared to the 40% reduction observed with GO/TiO_2_ surfaces. This suggests that Dox exhibited a synergistic effect with the GO material, efficiently inhibiting bacterial adhesion. This antibiotic acts by inhibiting bacterial protein synthesis [51] and likely makes cells more susceptible to the action of GO. Concerning biocompatibility, these materials did not adversely affect the viability of human fibroblasts, making them suitable for potential medical applications [48].

Other authors have modified GO surfaces with antimicrobial peptides (AMP), including the N-terminal fragment of Cathelicidin-2 (CATH-2) [49], ponericin G1 [8], or OH-CATH30 (OH30) [50]. In vitro results demonstrated that this association significantly impaired the growth of *Escherichia coli* and *Staphylococcus aureus* (up to 95% reduction). Furthermore, data from an in vivo study revealed that wounds containing AMP-GO materials exhibited six times fewer *S. aureus* cells than those containing AMP or the GN material alone [50]. The antimicrobial activity of AMP is well known, as they act by interacting with bacterial cell membranes, increasing their permeability and leading to cell death [49]. Thus, both AMP and GO target bacterial membranes, strengthening the antimicrobial action of the synthesized material. Additionally, these AMP-GO materials displayed low cytotoxicity towards mammalian cells (over 80% cell viability) [8,49,50].

Lastly, Lan et al. [36] developed an N-halamine-GO fibrous membrane, which was capable of inactivating *E. coli* cells by over 90%. N-halamines can transfer active halogen ions (e.g., Cl^+^) to bacteria through direct contact or release, thereby exerting antibacterial effects in combination with GO.

Although the materials mentioned above have been tested against a limited number of bacterial species (only *E. coli* and *S. aureus*), results suggest that they have promising antibacterial activity (over 90% biocidal activity) and excellent biocompatibility with human tissues.

## 3. Graphene Modified with Metals

Various metals and metal oxides have been utilized to modify the surface of GN and its derivatives in order to enhance their antimicrobial activity. Metals are known for their strong antimicrobial properties against a wide range of pathogens [52,53,54]. However, their biocompatibility can vary depending on factors such as the type of metal chosen and the method of conjugation. 

**Figure 3:**
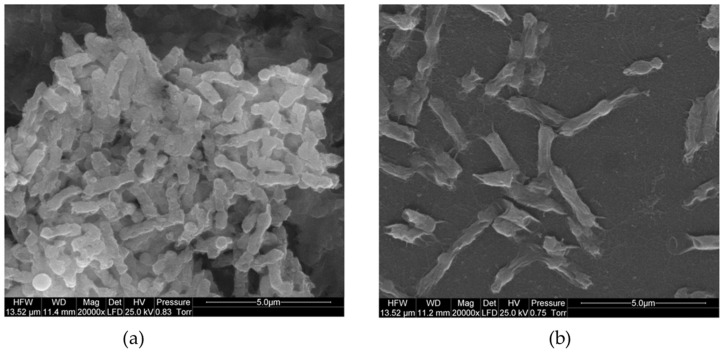
Scanning electron microscope (SEM) images of (**a**) *Salmonella enteritidis* bacteria and (**b**) *S. enteritidis* growing on silver nanoparticles (AgNPs)-graphene oxide (GO)-coated nanoplatform. Reprinted with permission from Ref. [55]. Copyright 2019 The Authors.

Table 2 presents studies that have evaluated the antimicrobial activity and biocompatibility of GN materials modified with metals or metal oxides. Several authors have focused on surface-modifying GO [55,56] or rGO [57,58] with silver nanoparticles (AgNPs). The resulting composites exhibited higher inactivation rates against both Gram-positive and Gram-negative bacteria, with the exception of the composite synthesized and tested by Wierzbicki et al. [55] against *Salmonella enteritidis* (approximately 50% reduction; Figure 3). The modification of GN-based materials with AgNPs results in a synergistic effect, as they inactivate bacteria by interacting with proteins and enzymatic thiol groups [57]. Furthermore, composites containing AgNPs appear safe for medical use.

The second most common metal used for GN surface modification is copper (Cu), either in the nanoparticle [54] or oxide form [52,53]. In general, Cu-GN materials demonstrated superior antimicrobial activity against Gram-positive bacteria (100% inactivation) compared to Gram-negative ones (20–90% inactivation), which can be attributed to the more intricate cell membrane structure of Gram-negative bacteria. The synthesized materials carry a positive charge that attracts bacterial membranes through physical adsorption and electrostatic interactions [52]. Additionally, they induced the generation of ROS, which ultimately kill bacteria. Furthermore, the developed materials exhibit excellent compatibility with human tissues [52,53]. 

The surface modification of GO with gold (Au) also exhibited promising activity against pathogenic bacteria (five-fold reduction) and did not affect the viability of human cells [59].

Although palladium (Pd)-reduced GO exhibited low biocompatibility, it is considered a promising material for tissue engineering, with bacterial inactivation ratios ranging from 72 to 90%, depending on the type of bacteria [60]. This composite wraps the bacterial cells and inhibits their metabolism. Lastly, cerium oxide (CeO_2_)-GO materials demonstrated moderate antimicrobial activity (30–40% bacterial inactivation) against both Gram-positive and Gram-negative bacteria by inducing ROS production [61]. 

Although they may be associated with lower biocompatibility, composites resulting from the combination of carbon materials and metals, particularly silver and copper, exhibit strong antimicrobial activity, mainly against Gram-positive bacteria. In addition, approximately 80% of the antimicrobial activity of developed GN–metal composites is attributed to GN action.

## 4. Graphene Modified with Polymers

Graphene surface modification with polymers is an interesting approach that can offer several advantages, including enhanced GN dispersion and improved mechanical properties and biocompatibility [62,63]. In addition, the association with polymers can potentially increase the antimicrobial activity of GN-based materials, as the polymers serve as a matrix that helps GN dispersion [35], thus promoting contact with microorganisms. Furthermore, some polymers have inherent antimicrobial properties because they contain cationic groups that can facilitate interactions with bacteria [64,65].

Table 3 summarizes studies addressing the biocompatibility and antimicrobial activity of GN materials modified with natural and non-natural polymers. Both pristine GN and GO have been modified with various polymers for potential medical applications (e.g., tissue engineering, wound dressing, or implantable medical devices). 

Hajduga et al. [64] produced polycaprolactone (PCL)-GN composites and evaluated their antimicrobial activity against Gram-positive and Gram-negative bacteria. While these composites were able to inactivate *S. aureus* by 90%, no effect was observed against *E. coli*, although both materials (GN and PCL) have known antimicrobial activity. These discrepant results may be related to the morphology of the tested bacteria, as previously described. In turn, poly(lactic-co-glycolic acid) (PLGA)-GN composites were developed and tested against *E. coli* under electric stimulation [9]. Results indicated that, at lower frequencies, synthesized films decreased bacterial viability by up to 60%. Lastly, Oliveira et al. [66] demonstrated that polydimethylsiloxane (PDMS)-GN composites significantly reduced the number of total (57%), viable (69%), culturable (55%), and viable but non-culturable (VBNC) cells (85%) of *S. aureus* biofilms, while a decrease of 25% in total cells and approximately 52% in viable, culturable, and VBNC cells was observed for *Pseudomonas aeruginosa* biofilms.

Concerning GO, its association with polyoxyalkyleneamine (POAA) [67] or poly(ε-caprolactone) (PCL) [68] resulted in composites that significantly decreased the viability of Gram-positive and Gram-negative bacteria (approximately 80%). Furthermore, a recently developed epoxy resin rich in GO demonstrated promising in vitro inactivation percentages against *E. coli* and *S. aureus* (57 and 97%, respectively) [69]. However, when evaluated in vivo, the antibacterial efficacy of this composite decreased to 47% for *E. coli* and 68% for *S. aureus*. 

**Figure 4:**
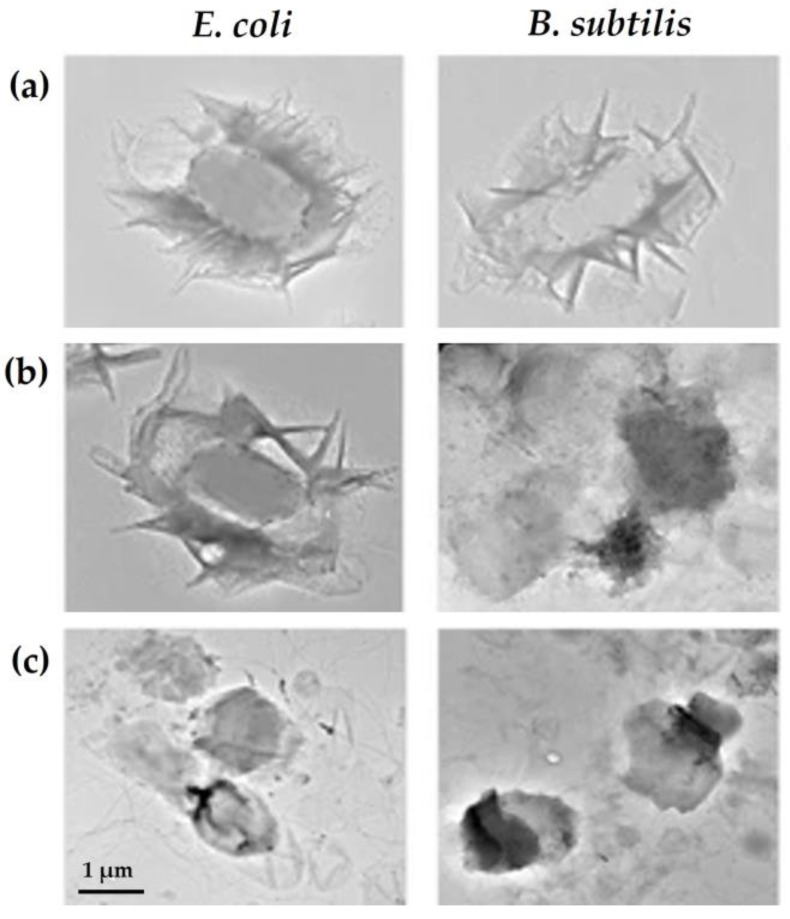
*Escherichia coli* and *Bacillus subtilis* exposed to (**a**) graphene oxide (GO), (**b**) polyoxyalkyleneamine (POAA)-GO, and (**c**) chitosan (CS)-GO for 3 h and characterized by transmission electron microscopy (TEM). Reprinted with permission from Ref. [67]. Copyright 2018 The Authors.

In vitro and in vivo studies have shown that GO surface modification with natural polymers, such as chitosan (CS) or carboxymethyl CS, increased its antimicrobial activity, resulting in over 90% inactivation of *Bacillus subtilis*, *E. coli*, and *S. aureus* cells [43,67], with no toxic effects observed in mammalian cells [43]. The combination of CS/poly(vinyl alcohol) with GO resulted in nanocomposites that could completely inhibit the growth of a wide range of pathogens, even at low concentrations (0.75 and 1 wt.%) [10,70]. Furthermore, biocompatibility assays demonstrated that these composites exhibited no toxicity towards pre-osteoblast cells, with over 70% cell viability [10]. Also, CS/polyethylene glycol-GO composites were promising for reducing *E. coli* and *S. aureus* viability (over 95% cell inactivation) while maintaining mammalian cell viability at 95% [42]. In fact, CS is known to be a powerful antibacterial compound that inactivates bacterial cells by interacting with their negatively charged membranes, leading to a decrease in their permeability and leakage of intracellular content. Additionally, CS can bind to bacterial DNA, thereby inhibiting the replication process. Lastly, CS is able to chelate metal ions which are essential for bacterial growth and proliferation [71]. Therefore, the surface modification of GN materials with CS enhances their interactions with bacteria, leading to more significant cell damage (Figure 4a,c).

Furthermore, the GO surface modification with folic acid and silk fibroin resulted in composites with high antibiofilm activity (80% inhibition of biofilm formation by *P. aeruginosa*) and biocompatibility (97% fibroblast viability) [71]. 

In general, these results indicate that the addition of GN materials to natural and non-natural polymers increased their antimicrobial activity by up to 70%, demonstrating a synergistic effect. Several authors have suggested that the main mechanism of action of polymer–GN composites is the wrapping of bacterial cells. When an external barrier made of GN-based materials is formed around the bacteria (Figure 4c) [67], it facilitates contact with cells, reduces access to essential nutrients for microbial growth, and induces oxidative stress, ultimately leading to cell death. Other authors have demonstrated that the effectiveness of polymer-containing composites results from a combined bactericidal and bacterial-repelling effect [72].

Regarding biocompatibility, human cells exposed to GN-based composites maintained their viability and proliferation capability [68,69]. In general, the combination of GN materials and polymers yielded composites with improved biocompatibility and substantial in vitro antimicrobial activity against medical pathogens.

**Table 3:** Studies reporting the biocompatibility and antimicrobial activity of graphene modified with polymers.

Graphene Material	Biomedical Application	Biocompatibility	Microorganism	Main Conclusions	Ref.
Non-natural polymers					
Polyoxyalkyleneamine (POAA)-graphene oxide (GO)	Surface coatings	NP	*Bacillus subtilis* *Escherichia coli*	After 3 h, bacteria exposed to POAA-GO decreased their viability to at least 75%.	[67]
Poly(ε-caprolactone) (PCL)-GO	Tissue engineering	Human fibroblasts kept their culturability and proliferation for up to 14 days.	*E. coli* *Staphylococcus epidermidis*	PCL-GO composites inactivated *S. epidermidis* and *E. coli* adhered cells by 80% after 24 h.	[68]
PCL-graphene (GN)	Nasal implants	NP	*E. coli* *Staphylococcus aureus*	The efficacy of PCL-GN against *S. aureus* was about 90%. In contrast, this composite did not exhibit activity against *E. coli*.	[64]
Epoxy-rich-GO (er-GO)	Wound dressing	Human cells exposed to er-GO exhibited viability ratios greater than 100%.	*E. coli* *S. aureus*	er-GO composite decreased in vitro *E. coli* and *S. aureus* viability by up to 57 and 97%, respectively. In vivo data indicated that *E. coli* and *S. aureus* viability was reduced by 47 and 68%, respectively, in presence of er-GO.	[69] *
Poly(Lactic-co-Glycolic Acid) (PLGA)-graphene nanoplatelets (GNP)	NE	NP	*E. coli*	At 15 MHz, PLGA-GNP composites reduced *E. coli* viability by 33%, while at lower frequencies (10 and 5 MHz), these films decreased bacteria viability by up to 60%.	[9]
Polydimethylsiloxane (PDMS)-GNP	Implantable medical devices	NP	*Pseudomonas aeruginosa* *S. aureus*	The PDMS-GNP reduced the number of total (57%), viable (69%), culturable (55%), and VBNC cells (85%) of *S. aureus* biofilms. A decrease of 25% in total cells and about 52% in viable, culturable, and VBNC cells was observed for *P. aeruginosa* biofilms.	[66]
Natural polymers					
Chitosan (CS)-graphene oxide (GO)	Surface coatings	NP	*B. subtilis* *E. coli*	After 3 h, bacteria exposed to CS-GO composite decreased their viability to less than 10%.	[67]
CS/poly(vinyl alcohol) (PVA)-GO nanocomposites	Tissue engineering	After 30 days of film implantation, the absence of injuries in the intervened areas with normal healing was observed.	*Bacillus cereus* *S. aureus* *E. coli* *Salmonella* spp.	Biocomposites containing 0.75 and 1 wt.% GO completely inhibited pathogen growth.	[70] *
CS/PVA-GO	Wound healing	CS/PVA-GO hydrogels showed nontoxicity towards pre-osteoblast cells (>70% cell viability).	*E. coli* *S. aureus*	Hydrogels exhibited high antimicrobial activity against *E. coli* and *S. aureus* (up to 35 and 32 mm inhibition halo, respectively).	[10]
CS/polyethylene glycol (PEG)-decorated GO biocomposite	Wound healing	Cell survival on CS/PEG-GO was 95.4%.	*E. coli* *S. aureus*	CS, 1 wt% CS/GO and 1 wt% CS/PEG-GO were able to inactivate *S. aureus* by 80, 85, and 100% and *E. coli* by 65, 85, and 95%, respectively.	[42]
Carboxymethyl Chitosan (CC)-GO-based Sponge	Wound healing	CC/L-cysteine-GO sponge showed a high cell viability rate, as demonstrated by Live/Dead staining.	*E. coli* *S. aureus*	In vivo data indicated that the CC/L-cysteine-GO sponge had a faster wound-healing rate than CC/GO. In vitro tests revealed that the addition of L-cysteine-GO and GO to CC increased sponges’ antimicrobial activity.	[43] *
Folic acid (FA)/silk fibroin (SF)-GO	Wound healing Tissue engineering	The viability of fibroblast cells exposed to FA/SF-GO for 24 h was 97%.	*P. aeruginosa*	After 24 h, FA/SF-GO film reduced biofilm formation by 80% compared to control (polystyrene).	[73]

NP, Not Performed; NS, Not Specified; VBNC, viable but non-culturable; *, in vivo study.

## 5. Graphene Modified with Natural Compounds

Owing to their biodegradability, renewability, and biocompatibility, there has been growing interest in composites that incorporate natural compounds. In recent years, several natural compounds, including vivianite [74], usnic acid (UA) [75], quercetin [76], and juglone [76], have been studied in conjugation with GN composites. Table 4 presents the biocompatibility and antimicrobial activity of these modified GN materials against several Gram-positive and Gram-negative bacterial species. 

Among the studies presented, natural compounds were mainly associated with graphene oxide.

**Figure 5:**
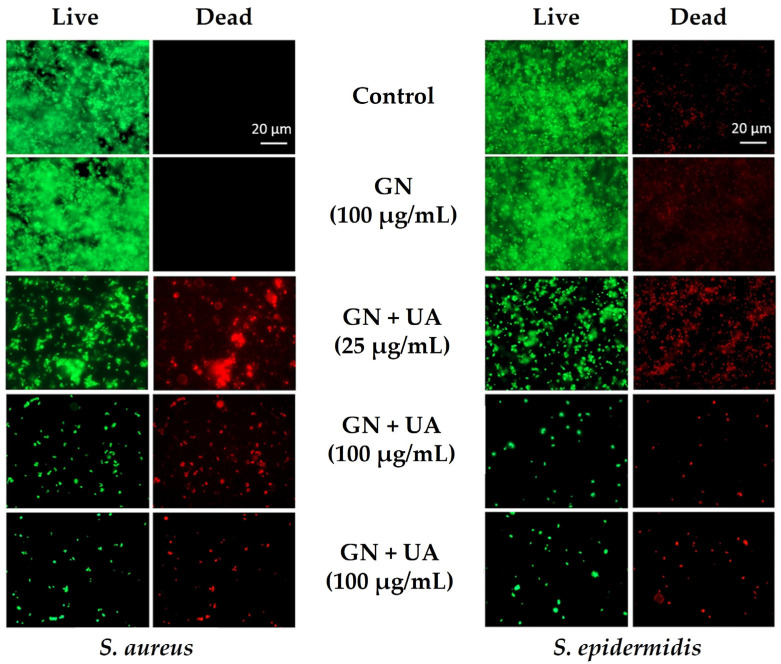
Antibiofilm capability of graphene (GN) and usnic acid (UA)-loaded graphene films against *Staphylococcus aureus* and *Staphylococcus epidermidis*. Representative fluorescence microscopic images from Live/Dead staining of staphylococcal biofilms. Reprinted with permission from Ref. [75]. Copyright 2021 The Authors.

Pandit et al. [75] conjugated GN with usnic acid and observed that, after 24 h of contact, the synthesized composites reduced the growth of *S. aureus* and *Staphylococcus epidermidis* by up to 3 Log in a dose-dependent manner (Figure 5). Furthermore, after 96 h, staphylococcal biofilms were reduced by 5 Log. The potent antimicrobial activity of UA is mainly based on the inhibition of RNA synthesis [75], which, in combination with GN antibacterial mechanisms, impairs bacterial growth. The hydroxyapatite/vivianite-GO composite also displayed promising antimicrobial activity against *E. coli* and *S. aureus* while preserving the viability of osteoblast cells [74]. The ferrous (Fe^2+^) and calcium (Ca^2+^) ions released from the composite may have contributed to potentiating its antimicrobial performance. Moreover, hydroxyapatite is well known for its exceptional biocompatibility. Croitoru et al. [76] modified GO surfaces with two natural antimicrobial agents, quercetin and juglone. Quercetin and juglone/GO composites reduced *S. aureus* culturability by 1 and 3 Log, respectively, and *E. coli* culturability by 5 Log. These GO-based materials showed biocompatible behavior at lower concentrations. 

Overall, natural compounds, particularly quercetin and juglone, enhanced the antimicrobial activity and biocompatibility of GN-based materials intended for medical purposes. 

## 6. Conclusions

This review comprehensively summarizes and discusses recently developed graphene-based materials, with a focus on their antimicrobial properties and biocompatibility, while exploring potential medical applications such as tissue engineering, medical devices, and wound healing. 

Biocompatible and antibacterial nanomaterials are in high demand for a variety of medical applications. The surface modification of GN and its derivatives (e.g., graphene oxide or reduced graphene oxide) with antimicrobials (e.g., antimicrobial peptides or biocides), metals or metal oxides, polymers, and natural compounds has enhanced the functionality and applicability of these materials, resulting in improved antimicrobial performance and increased biocompatibility towards human tissues. 

The combination of graphene materials with agents that possess intrinsic antimicrobial properties, such as antimicrobial peptides, metals (silver or copper), or chitosan, enhances the effectiveness of GN materials in inactivating bacteria, especially Gram-positive bacteria, because of their less complex membrane structure. Conversely, although promising for a wide range of applications, the use of non-natural polymers for GN surface modification results in composites with lower antimicrobial activity than those obtained through the modifications mentioned above. However, GN–polymer composites exhibit superior biocompatibility compared to antimicrobial or metal-based GN composites. In the latter case, adverse effects on human cells are highly dependent on the type of metal used and the methodology employed for their production.

Despite the significant progress made in these biomaterials, few in vivo studies have validated their effectiveness and applicability, which may hinder their translation into real medical scenarios. Additionally, studies addressing the long-term effectiveness, biocompatibility, and stability of these composites are lacking. Therefore, further research is needed to introduce these promising biomaterials in the medical field.

## Figures and Tables

**Table 1:** Studies focusing on the biocompatibility and antimicrobial activity of graphene modified with antimicrobials.

Graphene Material	Biomedical Application	Biocompatibility	Microorganism	Main Conclusions	Ref.
Doxycycline (Dox)-graphene oxide (GO) immobilized on titanium (TiO_2_)	Medical devices	Dox-GO/TiO_2_ did not affect the viability of human fibroblasts (over 80% cell viability).	*Escherichia coli* *Staphylococcus aureus*	Dox-GO/TiO_2_ reduced the viability of adhered bacteria by over 90%, whereas the GO/TiO_2_ surface inactivated adhered bacteria by 40%.	[48]
Antimicrobial peptide (CATH-2)–reduced graphene oxide (rGO)	Medical devices	Functionalized rGO induced low cytotoxicity towards erythrocytes in comparison to rGO alone.	*E. coli*	Peptide-functionalized rGO exhibited higher antimicrobial activity compared to rGO (13.3- and 21.8-mm inhibition halo).	[49]
Antimicrobial peptide (ponericin G1)/growth factor (bFGF)/poly(lactide-co-glycolide (PLGA)-GO composite	Wound healing	Produced composite increased cell proliferation compared to PLGA (*p* < 0.05).	*E. coli* *S. aureus*	Ponericin G1/PLGA-GO reduced bacteria growth compared to PLGA or PLGA-GO composite (*p* < 0.05).	[8]
Antimicrobial peptide (OH30)/polyethylene glycol (PEG)-GO	Wound healing	OH30/PEG-GO had high cell viability (over 80%) and low toxicity.	*S. aureus*	In vitro data demonstrated that OH30 released by the synthesized composite inhibited *S. aureus* growth by up to 95% after 3 h. In vivo data indicated that, on day 7, the number of *S. aureus* in wounds containing the composite was 6 times less than OH30 or PEG-GO (*p* < 0.05).	[50] *
N-halamine-GO fibrous membrane	NS	NP	*E. coli*	Synthesized composite exhibited high biocidal activity against *E. coli* (>90%).	[36]

NP, Not Performed; NS, Not Specified; *, in vivo study.

**Table 2:** Studies addressing the biocompatibility and antimicrobial activity of graphene modified with metals.

Graphene Material	Biomedical Application	Biocompatibility	Microorganism	Main Conclusions	Ref.
Silver nanoparticles (AgNPs)-reduced graphene oxide (rGO)	Medical textiles	NP	*Escherichia coli*	AgNPs-rGO composites exhibited enhanced activity against *E. coli* (100% inactivation) compared to rGO (82.5% inactivation).	[57]
AgNPs-graphene oxide (GO)	NE	The viability of human cells was not changed when incubated on nanoplatforms coated with AgNPs-GO.	*Salmonella enteritidis*	AgNPs-GO nanoplatform significantly inhibited *S. enteritidis* growth (over 50% cell inactivation).	[55]
AgNPs-rGO immobilized into polyurethane/cellulose acetate matrix	Wound healing	In vivo data demonstrated that AgNPs-rGO-based film significantly promoted the wound healing process.	*Pseudomonas aeruginosa* *Staphylococcus aureus*	The produced film exhibited an inactivation rate of 100% for Gram-negative bacteria and 95% against Gram-positive bacteria.	[58] *
AgNPs-GO deposited on nickel-titanium alloy	Medical devices	NP	*Streptococcus mutans*	AgNPs-GO reduced the number of *S. mutans* viable cells by up to 5 Log.	[56]
Gold (Au)-decorated amine-functionalized graphene oxide (NH_2_-GO)	Implant devices	Au-NH_2_-GO did not affect the viability of human cells (approximately 100% viability).	*Bacillus subtilis* *E. coli* *P. aeruginosa* *S. aureus*	The synthesized material exhibited a higher (5-fold more) antibacterial activity against Gram-positive and Gram-negative bacteria than bare Au or NH_2_-GO material.	[59]
Copper oxide (CuO)-GO nanohybrids into bacterial cellulose (BC) matrix	NS	CuO-GO/BC film exhibited excellent biocompatibility towards fibroblast cells (>100%).	*B. subtilis* *E. coli* *P. aeruginosa* *S. aureus*	After 3 h, CuO-GO/BC films completely inactivated Gram-positive bacteria while only reducing the viability of Gram-negative bacteria by 20%.	[52]
CuO-rGO	NS	NP	*P. aeruginosa*	CuO-rGO composites led to complete bacterial inactivation (7 Log reduction).	[53]
Copper nanoparticles (CuNPs)-graphene (GN) supported on silicon (Si) wafers	NS	CuNPs-GN/Si showed slight toxicity for human cells (15% reduction in cell viability).	*E. coli* *S. aureus*	In the presence of CuNPs-GN/Si films, *S. aureus* growth was completely inhibited, and *E. coli* viability was reduced by 87%.	[54]
Palladium (Pd)/polypyrrole (PPy)-rGO composite	Tissue engineering	Pd/PPy-rGO (<100 µg/mL) did not substantially affect osteoblast viability (>80%).	*B. subtilis* *E. coli* *Klebsiella pneumoniae* *P. aeruginosa*	Pd/PPy-rGO nanocomposite significantly inhibited the biofilm formation of *B. subtilis* (72%), *E. coli* (90%), *K. pneumoniae* (89%), and *P. aeruginosa* (83%).	[60]
Cerium oxide (CeO_2_)-GO	Wound healing	NP	*E. coli* *P. aeruginosa* *S. aureus* *Salmonella typhi*	CeO_2_-GO nanocomposite inhibited *E. coli*, *P. aeruginosa*, *S. aureus*, and *S. typhi* biofilms by 38, 40, 31, and 35%, respectively.	[61]

NP, Not Performed; NS, Not Specified; *, in vivo study.

**Table 4:** Studies demonstrating the biocompatibility and antimicrobial activity of graphene modified with natural compounds.

Graphene Material	Biomedical Application	Biocompatibility	Microorganism	Main Conclusions	Ref.
Hydroxyapatite/Vivianite-GO	NS	Cell viability of osteoblasts in the presence of this composite was 98%.	*E. coli* *S. aureus*	Composite exhibited activity against *E. coli* and *S. aureus* after 24 h (14.5 and 13.4 mm inhibition halo, respectively).	[74]
Usnic acid (UA)-GN	Medical devices	NP	*S. aureus* *Staphylococcus epidermidis*	After 24 h, UA-GN inhibited *S. aureus* and *S. epidermidis* biofilms by 3 Log at 25, 50, 100, and 200 µg/mL AU/GO compared to GN films and glass, except for *S. aureus* growing on 25 µg/mL AU-GN. After 96 h, staphylococcal biofilms were reduced by 5 Log compared to the control (glass).	[75]
Quercetin-GO	Drug delivery systems	GO-based materials showed a biocompatible behavior at lower concentrations (>70% cell viability).	*E. coli* *S. aureus*	Quercetin/GO composites reduced *S. aureus* culturability by 1 Log and *E. coli* culturability by 5 Log.	[76]
Juglone-GO	Drug delivery systems	Materials showed a biocompatible behavior at lower concentrations (>70% cell viability).	*E. coli* *S. aureus*	Juglone/GO composites reduced *S. aureus* culturability by 3 Log and *E. coli* culturability by 5 Log.	[76]

NP, Not Performed; NS, Not Specified.

## Data Availability

Not applicable.
